# Drink and Insanity

**Published:** 1898-08-13

**Authors:** 


### Modern Sociology.

### DRINK AND INSANITY.

Portion of a Paper by Dr. J. F. Sutherland, Read before the British Medical Association.

Dr. Sutherland said that inebriety, direct and collateral, next to heredity, and falling little short of it, was the most powerful agent in the production of insanity and imbecility, accounting proximately and indirectly for 25 per cent. of those who passed within the portals of asylums, and for a deal of insanity unknown to the local or central authorities. Some might think the estimate of 25 per cent. too high, but it would be much higher were he to include as insane those who were the victims of dipsomania, mania-à-potu, and delirium tremens, and possibly reach 35 per cent. His investigations into the problem, extending over many years, led him to the conclusion that inebriety, whether criminal or non-criminal, was a disease or vice, or both; for the vice long indulged ultimately ended in functional and structural changes, and was in the main, like insomnia, neurasthenia, neuralgia, and hysteria, met with in large centres of population, and presumably was in great measure due to unhygienic and uncomfortable surroundings, to the facilities for illicit sale, to vicious and contaminating environments, to customs and habits long practised in certain strata of society, to vocations implying hard, unremitting, and, it might be, uncongenial toil, and to the worries, struggles, and disappointments incidental to all such communities. It was said the habitual inebriate was violating no law of the land. True, but were they justified in allowing him to go on until he committed a crime of the first magnitude, which many of them did, and many more by the merest accident did not do? It was too late to deal with them after a crime was committed. Legislation, to be fair and just, should also be preventive. The liberty of the subject had with a few become a fetish. A law which would prevent and lessen the physical and moral degradation of the individual, the wreckage of family life, and what was a disgrace both to the community and to the commonwealth, could in no reasonable sense be considered an invasion of liberty. The present voluntary system of entering retreats would continue, and the French system of "conseil de famille" might be introduced to our legislation as something midway between voluntary submission and compulsion. The insanities of inebriety numbered seven: (1) Intoxication; (2) delirium tremens; (3) mania-à-potu; (4) dipsomania; (5) monomania of suspicion; (6) chronic alcoholism or dementia; (7) general paralysis. The number who suffered from the last three might be estimated, as, as a rule, they found their way to asylums. Not so those from the first four, whose numbers at least reached to four figures. In regard to the six insanities the law had looked upon all, save delirium tremens, dipsomania, and intoxication, which was a transient and temporary insanity, as an excuse for criminal acts; and in later years two of the most eminent judges who had adorned the Scottish Bench—the late Lord President Inglis and the late Lord Deas—and others put delirium tremens in the same category as the other insanities. The position of dipsomania was not quite so satisfactory; but it might be taken from the ruling of Lord Young in 1893, when a dipsomaniac was tried for the neglect and starvation of her infant child, that it likewise would be a good answer to a charge as barring trial or excusing the offender. But the criminal law of this country—and in this respect our position was exceptional among civilised nations—was far astray in regard to its attitude towards the authors of homicide, assaults, &c., committed by persons in a state of intoxication; not so, however, the interpretation of it, for some judges in England and Scotland had seen their way to reduce the crime of murder to manslaughter or culpable homicide. But this was not the attitude of all, and so they would like to know where they stood in this matter. Intoxication was insanity; the civil law in this country had looked upon it as insanity, or something akin to it, and as nullifying contracts, marriages, testamentary deeds, dispositions, &c. Why in reason should there be such a difference between the attitude of the civil and criminal laws? Except the homicides and assaults of homicidal maniacs, 95 per cent. of the homicides and assaults of our criminal calendars were committed by persons in a state of intoxication, and some such had forfeited their lives for their crimes. Some judicial fallacies—if he might venture, with all respect, to say so—had grown round this question. The drinking was said to be voluntary, the act deliberate, and the death sentence a deterrent to drunkards and habitual drunkards, and a protection to society. It was no more the latter than in the past times the gallows was to sheep stealers, and excommunication and burying of drunkards in the public highway to the drunkards of last century. And yet only a few days ago it was seriously advocated that the therapeutics of drunkenness should include the lash and cautery. Parliament in its wisdom, six years ago registered the high-water mark of a matured public opinion as to the punishment it would sanction. The drunkard was allowed by the laws to drink to excess, drunkenness *per se* was not an offence, and no hand lifted, although he was known to be a noxious and potentially dangerous subject in the community, until he committed a crime of the first magnitude. Society would be protected by putting the inebriate criminal in "a safe place of custody," and not holding him responsible for an act of which he had, on regaining sobriety, either no real consciousness or the dimmest. There was no evidence forthcoming in support of the theory that the gallows was a deterrent to drunkards. Assaults were as numerous as ever, and it was the merest accident many more of them did not terminate fatally. The violence used was not, indeed could not, be measured. The savage and brutal nature of such crimes was indicative not of premeditation, but rather of blind and furious mania. His own feeling was that the plea of insane at the time was a proper special defence, or, failing the acceptance of that, that the crime, after medical and other evidence had been led, should be reduced from murder to culpable homicide. This was all the more reasonable, having regard to the present state of the law, which ignored overt drunkenness and habitual drunkenness, and this other, that the defence of such persons indicted for grave crime was often conducted without the aid of the specialist, and without any, or a moiety, of the resources at the back of the public prosecutor.

